# Interstitial Photodynamic Therapy—A Focused Review

**DOI:** 10.3390/cancers9020012

**Published:** 2017-01-24

**Authors:** Gal Shafirstein, David Bellnier, Emily Oakley, Sasheen Hamilton, Mary Potasek, Karl Beeson, Evgueni Parilov

**Affiliations:** 1 Photodynamic Therapy Center, Department of Cell Stress Biology, Roswell Park Cancer Institute (RPCI), Elm & Carlton Streets, Buffalo, NY 14263, USA; David.Bellnier@RoswellPark.org (D.B.); Emily.Oakley@RoswellPark.org (E.O.); Sasheen.Hamilton@RoswellPark.org (S.H.); 2 Simphotek, Inc., 211 Warren St, Newark, NJ 07103, USA; mpotasek@simphotek.com (M.P.); beesonk@simphotek.com (K.B.); evp@simphotek.com (E.P.)

**Keywords:** interstitial photodynamic therapy, prostate, head and neck, pancreatic, brain, treatment planning

## Abstract

Multiple clinical studies have shown that interstitial photodynamic therapy (I-PDT) is a promising modality in the treatment of locally-advanced cancerous tumors. However, the utilization of I-PDT has been limited to several centers. The objective of this focused review is to highlight the different approaches employed to administer I-PDT with photosensitizers that are either approved or in clinical studies for the treatment of prostate cancer, pancreatic cancer, head and neck cancer, and brain cancer. Our review suggests that I-PDT is a promising treatment in patients with large-volume or thick tumors. Image-based treatment planning and real-time dosimetry are required to optimize and further advance the utilization of I-PDT. In addition, pre- and post-imaging using computed tomography (CT) with contrast may be utilized to assess the response.

## 1. Introduction

In photodynamic therapy (PDT) visible or near-infrared light is used to activate a light-sensitive drug (photosensitizer, PS) that, in the presence of ground state oxygen, creates reactive oxygen species and radicals that can induce tissue death [1,2]. Most often, external beam PDT (EB-PDT) is used to treat superficial lesions, where the effective depth of light penetration and treatment is limited to <10 mm [3,4]. Intra-tumor light delivery (interstitial PDT, I-PDT) is required to activate PS in deeply seated tumors or tumors that are more than 10 mm in thickness.

**Figure 1:**
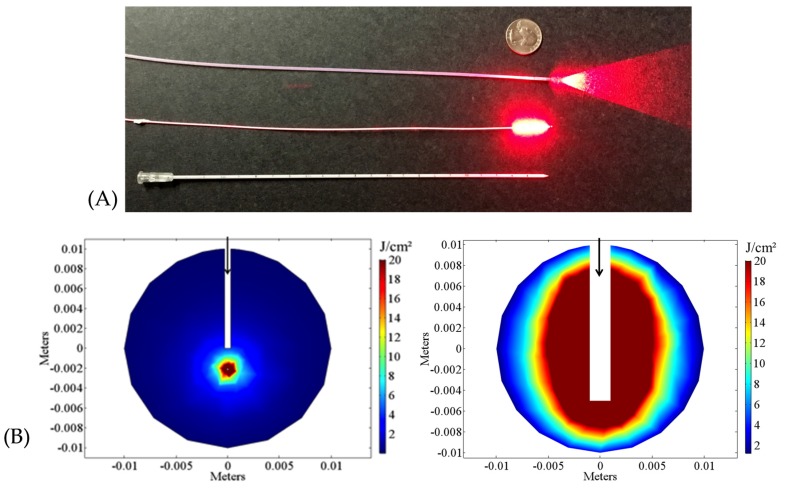
Laser treatment fibers. (**A**) A typical 600-µm-diameter flat-cut (top), a 0.98-mm-diameter 1-cm cylindrical diffuser fiber emitting red laser light (middle), and an optically transparent plastic catheter with a 2-mm-outer diameter and a 1.45-mm-inner diameter (bottom) that can be used for insertion of cylindrical diffusers [10]; (**B**) Computer simulation of light propagation from a flat-cut fiber (bottom left), and a 1-cm cylindrical diffuser (bottom right) in a catheter. The black arrows point to the location of the fibers in a 2-cm-diameter spherical geometry with optical properties set to be similar to those measured for 652-nm light in a head and neck tumor by Robinson et al. [12]. A detailed description of the mathematical model and basic assumptions are given in Shafirstein et al. and Oakley et al. [13,14]. In interstitial photodynamic therapy (I-PDT), the light energy density (J/cm^2^) or light dose is calculated as dose volume histogram (DVH), which is the minimum light dose absorbed in a certain percentage (typically 90%) of the target volume [15].

In I-PDT, one or more laser fibers are inserted into the target tissue, typically tumor and margins. The laser fibers can be inserted via needles, or placed in catheters [5]. The light can be delivered through the end of the fiber (flat-cut) fibers, or through a fiber with a cylindrical diffuser end (as shown in Figure 1A). In utilizing a flat-cut fiber, a needle is inserted to required depth, the fiber is passed through to the end, then the needle is pulled back to expose the tip of fiber in the tissue [6,7]. Typically, fibers with a cylindrical diffuser end are inserted into optically transparent catheters that are placed into the tumor [8,9,10]. The cylindrical diffuser end can be of various lengths, ranging from 0.5 cm to 7 cm (Medlight, Ecublens, Switzerland; Pinnacle Biologics, Bannockburn, IL, USA; Biolitech, Cerm Optec, Bonn, Germany). The number and location of the treatment fibers vary according to tumor size and location, anatomy, PS and the light wavelength used to treat. The light distribution around the fibers depends on the fiber type. Recently, computer simulations suggested that cylindrical diffuser fibers are more effective than flat-cut fibers in delivering the therapeutic light [11]. However, both fibers are being used in clinical settings. In Figure 1B we illustrate the differences in light distribution between a flat-cut and a cylindrical diffuser placed within a spherical geometry.

Many studies have employed I-PDT in the treatment of prostate, pancreatic and head and neck cancer, as well as esophageal, brain and other deeply-seated and locally-advanced cancerous tumors. This review is focused on I-PDT, and its applications in prostate, pancreatic, head and neck and brain cancers. In I-PDT, image-based treatment planning is required for administering the therapy. Hence, this review will include reference to image-based treatment planning. The overall objectives are to highlight the progress and suggest new directions that could advance the utilization of I-PDT.

## 2. Interstitial PDT (I-PDT) in Prostate, Pancreatic, Head and Neck, and Brain Cancers

### 2.1. I-PDT in Prostate Cancer

The standard of care (SOC) treatment options for prostate cancer are surgery and radiation therapy. I-PDT has been evaluated as an alternative therapy in patients with early stage, localized and recurrent prostate cancer [16,17,18,19]. These studies showed that I-PDT is associated with minimal side effects (compared to surgery and radiation) and can be repeated several times for disease control. Table 1 presents a summary of clinical studies conducted in the past 15 years that utilized I-PDT with second generation PS´s including: 5-aminolevulinic acid (ALA), motexafin lutetium (MLu, Lutex), palladium bacteriopheophorbide (Tookad^TM^, Steba Biotech, Luxembourg, Luxembourg) and meso-tetrahydroxyphenylchlorin (mTHPC, Foscan^TM^, Biolitec Pharma Ltd., Dublin, Ireland). The PS is administered to the patient systemically prior to the light illumination. After a few hours or days, depending on the PS, the I-PDT treatment (light delivery) commences when maximum tumor/skin ratio of the PS is expected. Image-based planning is accomplished with three-dimensional (3-D) model of the prostate and surrounding tissues that is reconstructed from transrectal ultrasound (TRUS) images. A transparent template is used to position hollow plastic brachytherapy-like catheters into the prostate. The template provides a grid of possible catheter positions separated by 0.5 cm in lateral and vertical directions. In most cases, cylindrically-shaped diffusing optical fibers are inserted into the catheters whose number and position are adapted to each patient according to the tumor size. Laser light is transmitted to the tissue through the optical fibers. The exact wavelength varies depending on the absorption of the drug. Various values for the energy (J), energy density (J/cm^2^), linear energy (J/cm) or intensity (mW/cm) are listed in Table 1.

The techniques used to analyze the treatment outcomes include: prostate-specific antigen (PSA) test, necrosis, and blood oxygenation level-dependent (BOLD)-contrast magnetic resonance imaging (MRI) [20]. In many cases, BOLD-contrast MRI is applied before, during, and after I-PDT of the prostate tumor.

#### 2.1.1. Image-Based Treatment Planning in I-PDT of Prostate Cancer

Davidson et al. developed a treatment-planning software package and employed it in a Phase II clinical trial of Tookad^TM^-mediated I-PDT of persistent prostate carcinoma following radiation therapy [15]. This software used a patient specific I-PDT treatment planning based on predicted light distributions in the prostate and surrounding tissue. The model used the diffusion equation and the finite elements method (FEM) numerical analysis with the volume of interest discretized into a 4-noded tetrahedral mesh. Treatment plans were based on pre-treatment MRI images. Optical properties were determined by fitting a diffusion-based model to the in vivo fluence rate measurements. The treatment plan was evaluated by the light dose distribution superimposed on the MRI images of the largest volume. Light distribution calculations were verified by comparing fluence rate measurements made prior to Tookad^TM^ infusion, with fluence rate data extracted during treatment. Treatment results were measured 6-months post-treatment with biopsies. In tumors treated with light dose greater than 23 J/cm^2^, a complete pathological response was observed in biopsies collected from the treated tumor. The dosimetry concentrated on the light optical properties and the light fluence delivered to various regions of the prostate.

Spectracure developed a treatment planning software, Interactive Dosimetry by Sequential Evaluation (iDOSE) that provided dose plans with optical fiber positions based on 3-D tissue models generated from ultrasound [30]. The software calculates the best fiber positions and provides an optimal plan. A first monitoring sequence is performed after the optical fibers are in place. Initially, homogeneous optical properties are assumed for each cluster of optical fibers and initial monitoring is performed. Based on the diffusion approximation, an initial calculation of the light dose is performed and effective attenuation coefficients are determined. At specific intervals the light is interrupted and a monitoring evaluation test is performed. Tissue optical properties were obtained using the same fibers used for delivering the therapeutic light. This enables one to determine the effective attenuations and update the light dose. In a Phase I/II clinical study, the Spectracure system was used to administer I-PDT with mTHPC in the treatment of patients with histologically-proven, untreated, organ-confined prostate cancer. Initially a conservative light dose of 5 J/cm^2^ was used to limit damage to surrounding tissue. Following I-PDT, the PSA level was higher than expected for complete treatment and it was concluded that the light dose of 5 J/cm^2^ was insufficient. In a later pre-clinical study in male canines, this group suggested that the threshold light dose should be in the range of 20–30 J/cm^2^ for effective I-PDT with mTHPC in the treatment of prostate cancer [31].

Several preclinical and clinical studies on prostate I-PDT have been carried out at the University of Pennsylvania [22,32,33]. In a Phase I trial seventeen patients were treated with MLu [33]. Cylindrical diffusing fibers were used as light sources placed about 1 cm apart. About 24 h before treatment, 0.5 to 2 mg/kg MLu was administered intravenously. A diode laser operating at 732 nm was used with fluences from 25 to 150 J/cm^2^. Fluorescence measurements were used to determine the MLu concentration in the tissue. PSA levels were scheduled at 2 weeks after discharge, monthly for 3 months, then every 3 months for up to 2 years. The data showed that higher I-PDT dose (i.e., MLu concentration times light fluence) led to greater increases in PSA at 24 h after I-PDT (119% versus 54%) suggesting that higher I-PDT dose creates greater tissue damage. The data in this study suggested that PSA changes could provide useful information about the tissue effects of I-PDT treatment in prostate, and post-treatment PSA levels may identify patients’ suitability for biopsy.

#### 2.1.2. Phase III Trial of PDT versus Active Surveillance of Prostate Cancer

Recently a Phase III I-PDT trial was performed using Tookad^TM^ in a randomized controlled trial at 47 EU university centers and hospitals on men with low-risk, localized, prostate cancer [27]. There were 206 patients for PDT and 207 for active surveillance. Active surveillance delays intervention for low-risk prostate cancer in order to prevent overtreatment. An advantage of both treatments is the increase in tissue preservation relative to other treatments, which can substantially improve the quality of life for patients. The outcome of that Phase III trial was encouraging, with negative biopsy after 24 months for 101 (49%) prostate cancer patients with I-PDT and only 28 (14%) of prostate cancer patients with active surveillance. The results of this multicenter study show that I-PDT using Tookad^TM^ can be used widely and effectively in low-risk, localized, prostate cancer. While this study is promising, the authors cite the need to investigate long-term effects and the survivability of the surrounding tissues. However, a significant advantage of this I-PDT treatment is the tissue preservation of the patient.

### 2.2. I-PDT for Pancreatic Cancer

In preclinical work on pancreatic cancer, Samke et al. studied pancreatic cancer xenograft models by injecting human pancreatic cancer cells into the pancreases of immunocompromized mice [34]. Two types of human cells were used, AsPC-1 and Panc-1. AsPC-1 injections resulted in rapidly-growing tumors that reached volumes of 60 mm^3^ in 2 weeks and had large, chaotic, and highly perfused blood vessels. Panc-1 injections resulted in slower growing tumors that reached 60 mm^3^ volumes in 5 weeks and contained smaller, organized blood vessels with less perfusion. Tumor total volumes and tumor vascular perfusion volumes were measured by MRI 24–48 h pre- and 48 h post-I-PDT. After the tumors each reached 60-mm^3^ total volume, I-PDT was performed on each mouse using a diffusing fiber (320-µm-core diameter, 1-cm-long diffuser tip) and 690-nm laser light at a linear irradiance of 74 mW/cm. The escalating light dose treatment plan had four groups (three mice each) for each tumor type: 10 J/cm, 20 J/cm and 40 J/cm with verteporfin, and 40 J/cm without verteporfin. Following the 48-h, post I-PDT MRI, the mice were euthanized. The tumors were excised, fixed and sectioned for fluorescence (using 1.0 mg/kg 3,30-diheptyloxacarbocyanine iodide DiOC7(3)) and histological analysis. The fluorescence data were used to sample the number of blood vessels in each tumor following treatment.

The AsPC-1 and Panc-1 tumors had very different responses to I-PDT. Both tumor types had low response to 10 J/cm. Both types responded to 20 J/cm and to a greater extent at 40 J/cm. Total tumor volumes and vascular perfusion volumes increased as light dose was increased. Volumes in control groups exposed at 40 J/cm without verteporfin were similar to volumes before I-PDT. The increase in total volume after verteporfin I-PDT is likely due to acute inflammatory response to I-PDT. Total volume, vascular perfusion volume and non-perfusing volume (non-perfusing volume is total volume minus perfusing volume) increased more rapidly for AsPC-1 and peaked at 20 J/cm. The greatest volume for Panc-1 was reached at 40 J/cm and the resulting total volume was approximately the same as for AsPC-1 at 20 J/cm. The size of the necrotic region increased more rapidly with light dose for AsPC-1 compared to Panc-1, indicating a different response for the two tumor lines for identical treatments. Doses of 20 J/cm and 40 J/cm resulted in a decrease in the number of blood vessels after treatment and an increase in non-perfusing volume. For AsPC-1, a dose of 40 J/cm resulted in complete necrosis of the tumor plus some necrosis of the surrounding pancreas, indicating that 40 J/cm was too high for the faster growing AsPC-1 tumors. This was expected since AsPC-1 tumors have higher levels of vascular endothelial growth factor and epidermal growth factor as well as larger ill-formed blood vessels. For both tumor types, increasing dose resulted in a decrease in the number of blood vessels. At 40 J/cm, no blood vessels remained in the AsPC-1 tumors. The overall results indicate that for a fixed I-PDT treatment light dose, one can expect variations in pancreatic cancer treatment outcomes that depend on specific tumor characteristics.

In addition to the preclinical work described above, two representative clinical trials have been performed that are listed in Table 2. An additional follow-up study was done on the results of the second trial.

Bown et al. conducted a phase I study of I-PDT in the United Kingdom for 16 patients with inoperable pancreatic cancer [6]. Tumor diameters varied from 2.5–6.0 cm (median 4.0 cm) and tumor volumes were 3–63 cm^3^ (median 27 cm^3^). Computed tomography (CT) contrast scans were used to guide fiber insertions during I-PDT. Patients were administered 0.15 mg/kg mTHPC intravenously, three days prior to the light delivery. Depending on the size of the tumor, up to six needles were inserted into each patient (percutaneously) to the required depth by a radiologist with CT guidance. The needle tips were separated by approximately 1.5 cm. Optical fibers with 0.4-mm cores were inserted into the needles through to the ends of the needles, and then the needles were partly withdrawn such that 3 mm of bare fiber extended from each of the needles and was in direct contact with the tumor. Red light from a 652-nm diode laser was directed through a beam splitter and was calibrated so that each fiber delivered 100 mW to each fiber tip, and the needles were pulled back in steps of 1 cm under CT control to deliver 20–40 J. Follow-up contrast-enhanced CT scans were performed on each patient a few days after I-PDT. The I-PDT-induced necrosis was visible as dark areas (less contrast enhancement) in the CT images. The total volume of necrosis varied from 9–60 cm^3^ (median 36 cm^3^). The volume of necrosis around each individual fiber ranged from 1.4 to 5.1 cm^3^ (median 2.9 cm^3^). The variation may be due to variations in PS concentration or to a small amount of blood at the fiber tip reducing light transmission. The authors suggest it may be necessary to monitor PS levels and light intensity in the tissue during I-PDT. Long-term patient monitoring was also done and included CT scans. The tumors did not regrow in the necrotic areas but did regrow from the edges of treated areas. Most patients had poor exocrine pancreatic function before I-PDT, which was worse after I-PDT and required pancreatic supplements. Survival time after I-PDT ranged from 4 to 30 months (median 9.5 months), which is comparable to other types of treatments. The I-PDT treatment resulted in a shorter recovery time than pancreatic resection and can be repeated if needed. The main shortcomings of Foscan^®^-mediated I-PDT are that injections must be made three to four days before administering the treatment light, and those patients remain sensitive to direct sunlight for about one month.

Additional studies of I-PDT for pancreatic cancer have been done using verteporfin (Visudyne^TM^, Valeant Pharmaceuticals, Bridgewater, NJ, USA). The verteporfin is a vascular-targeted PS, and therefore has a very short metabolic half-life compared to mTHPC. Verteporfin can be administered just one hour before light treatment and patients are light sensitive for only 24 h after treatment. Verteportfin causes damage to blood vessels as well as cell necrosis from singlet oxygen. The drug has been approved in the U.S. for treating age-related macular degeneration.

Huggett et al. describe the results of a Phase I/II trial of verteporfin I-PDT on locally advanced pancreatic cancer that was sponsored by the University College London [35]. Fifteen patients with locally advanced cancers in the head of the pancreas and who could not undergo surgical resection were given 0.4 mg/kg verteporfin. A single hollow metal needle (13 patients) or multiple needles (2 patients) were inserted into the tumors (percutaneously) using CT guidance. A fiber diffuser (0.4-mm-core diameter, 10-mm-long diffuser tip) was inserted into each needle and the needle pulled back to expose the 10-mm fiber diffuser, which was positioned directly in contact with the tumor. A 690-nm, 0.3-W diode laser was calibrated to deliver 150 mW/cm along the diffuser tip. Treatments began 60–90 min after the verteporfin was administered. Using single fibers, the treatment plan consisted of delivering increasing light doses (5 J/cm, 10 J/cm, 20 J/cm and 40 J/cm) to four groups of three patients each. One patient was treated with two fibers with a light dose of 40 J/cm for each fiber. Another patient was treated with three fibers. Comparing CT scans before and after treatment monitored the I-PDT-induced necrosis and tumor volumes. No necrosis was observed in patients treated with 5 J/cm. At 10 J/cm, one patient had a necrosis diameter over 12 mm. At 20 J/cm, necrosis diameter was over 12 mm in two patients. At 40 J/cm, necrosis diameter was over 12 mm for all three patients. Necrotic volumes were determined for all patients and generally increased with increasing light dose. However, there was considerable variation within each light dose group, making it difficult to predict necrosis diameter based on just the PS dose, the drug–light interval, and the light dose. The authors suggest the variations may be due to differences in the pharmacokinetics of the PS between patients or to variations in tissue and vascular diffusion that influence light penetration. The median survival after I-PDT was 8.8 months and was comparable to patients undergoing conventional treatment. The median survival from diagnosis was 15.5 months.

Jermyn et al. did a follow up analysis of the Phase I/II trial described above using CT contrast data [36]. Approximately 60–90 min prior to I-PDT treatments, high-resolution contrast and non-contrast CT scans were done to determine the arterial and venous blood content of the pancreas tissue and the blood vessels. Venous blood volume data before treatments were then compared to necrotic volumes following verteporfin-based I-PDT. The volume of necrosis is defined by a sharp boundary due to an I-PDT threshold effect. Necrotic volumes were normalized by the energy delivered and were compared to venous blood content before I-PDT. There was a very high negative correlation (R^2^ = 0.85) between the parameters (i.e., higher venous blood content resulted in lower normalized necrotic volume). There was a low correlation to arterial blood content (R^2^ = 0.22). Venous blood has a higher percentage of deoxy-hemoglobin than arterial blood and deoxy-hemoglobin has a higher optical absorption than oxy-hemoglobin at the therapeutic wavelength used. Therefore, a higher concentration of deoxy-hemoglobin will lead to higher optical absorption, lower light penetration into the tissue and a smaller necrotic volume. The results suggest that light attenuation is the dominant factor in I-PDT treatment response. Necrotic volumes resulting from I-PDT have been difficult to predict due to a lack of information about in vivo tissue optical properties. The authors suggest that light modeling has the ability to estimate necrotic volumes and that contrast CT can assist in pre-treatment planning for I-PDT of pancreatic cancer.

### 2.3. I-PDT for Locally-Advanced Head and Neck Cancer

I-PDT with mTHPC has shown promising results in the treatment of patients with locally-advanced head and neck cancer (LAHNC) that failed to respond to standard therapies [37,38].

In a pivotal Phase II study, 45 patients with LAHNC that failed radiation and chemotherapy were treated with I-PDT with mTHPC (Foscan^®^, Biolitec Pharmaceuticals Ltd., Dublin, Ireland) [37]. MRI or CT was used for imaging the tumor and determining the placement of the catheters. A single drug dose of 0.15 mg/kg Foscan^®^ was administered intravenously, four days prior to I-PDT. A total of 67 treatments were administered to 45 patients. A light dose of 20 J/cm was delivered at 100 mW/cm through flat-cut laser fibers inserted through 18 gauge needles, at about 15 mm apart. The needles were inserted through the oral cavity skin or both in 36 (54%), 25 (37%) and 6 (9%) treatments, respectively. The authors reported that of the treated tumors, twelve of 45 (27%) were close to major structures deep in the neck (e.g., carotid artery), another nine of 45 (20%) had invaded up under the base of skull, while seven of 45 (16%) had compressed the trachea. A serious complication was a carotid rupture 2 weeks after I-PDT in two patients, where the tumor invaded the carotid artery. Therefore, tumor invasion of any major blood vessel is a contraindication for I-PDT. No loss of function was detected in nerves encased by treated tumors. Nine patients (20%) achieved a complete response, of which six (13%) had no evidence of recurrence at their last follow-up (13–60 months). Eight of the nine complete responders were evaluated for one-year survival; all eight (18% of 45) survived more than a year, of which four were alive and disease-free (13–60 months). Another 24 subjects of 45 (53%) achieved worthwhile palliation of symptoms, of which 8 of 45 (18%) survived more than a year, including 2 still alive at the time of publication (survivals of 24 and 31 months). The median survival was 14 months overall, divided as 16 months for the 33 responders (73%) versus 2 months for the 12 non-responders. Consequently, the European Medicines Agency (EMA) granted an approval for I-PDT with Foscan^®^ for the treatment of patients with refractory LAHNC.

Several other groups reported the results of prospective and retrospective studies utilizing I-PDT with Foscan^®^ in the treatment of LAHNC in the European Union [7,8,12,38]. Jager et al. utilized MRI guidance for fiber insertion into the target tumors [38]. They treated 14 patients and achieved median survival of 14 months as reported in Lou et al. 2004 [37]. No specific treatment plan computation was used, but they attempted to keep the distance between the fibers at about 15 mm. Jerjes et al. utilized intraoperative ultrasound guidance for I-PDT in the treatment of 21 patients with LAHNC [7]. Pre-treatment MRI was used to assess tumor volume. An 18-gauge, 70-mm-long spinal needle was used to place the 400-μm-core diameter flat-cut polished fibers into the tumor. The treatment fibers were inserted in the needle and allowed to protrude by 2–3 mm from the end of the needle. The needles were pulled back in increments of 10 mm, within the target tumor. Ultrasound imaging was used to guide the placement and the position of the fibers during the pullback. The radial distance between adjacent needles was approximately 7 mm. No computerized planning was employed.

Karakullukcu et al. used modified brachytherapy techniques for treatment planning for I-PDT in the LAHNC [8,39]. The plan has been shown useful in assisting physicians in decision making with respect to how many fibers to place, but it does not calculate light fluence distribution.

Baran and Foster developed a treatment planning that uses graphics processing unit-enhanced Monte Carlo (MC) simulations to model the delivery of light in near-real time in tissue volumes representing head and neck tumors [11,40]. They use their model to compare treatment time and light dose when the light is delivered from a flat cleaved fiber or cylindrical diffuser fibers. Their analyses suggest that the cylindrical diffuser are more effective than the flat cleaved fibers [11].

Oakley et al. published a treatment planning using FEM to simulate light propagation in geometries that accurately mimic LAHNC [14]. This study demonstrated that FEM could be utilized to simulate light propagation in large and complex head and neck anatomy. The authors also showed that the computation time could be reduced to less than 4 min by optimizing the mesh of the FEM model.

A summary of the main studies in I-PDT of head and neck cancer is provided in Table 3.

### 2.4. I-PDT for Brain Cancers

Malignant brain tumors can be highly invasive and are difficult to treat. Malignant gliomas account for 1% of all worldwide cancer tumors but result in 2% of cancer deaths. Typical post-diagnosis survival times are approximately 16 months using standard treatments of surgical resection, radiation and chemotherapy [41,42].

Recent I-PDT clinical studies of malignant gliomas are very limited. Most PDT studies done between 1980 and 2006 utilized intracavitary PDT following resection [41]. Resection has the advantage of removing most of a tumor before adjuvant therapies such as PDT, chemotherapy and radiation therapy to reduce the recurrence of tumors at the margins of the resultant cavity. In some of the early work, I-PDT supplemented the intracavitary PDT [43]. Two recent small I-PDT studies are listed in Table 4.

Beck et al. [44] did a limited I-PDT study using 5-ALA-induced protoporphyrin IX on 10 patients where malignant gliomas had recurred. The maximum diameter of the tumors was 3 cm. Patients received 5-ALA orally at 20 mg/kg body weight one hour before light treatment. Light was supplied by a 4-watt, 633-nm diode laser that was split into up to six beams. Each beam was transmitted by an optical fiber to a cylindrical light diffuser with an outer diameter of 1.6 mm and a length of 20 mm or 30 mm. Imaging for treatment planning combined data from CT scans, MRI scans and positron emission tomography (PET) scans to visualize the tumor and to control placement of the diffusing light sources. The irradiation time was 1 hour with a light power to each diffuser of 200 mW/cm diffuser length. The light fluence per diffuser length was 720 J/cm and the total light applied to a tumor ranged from 4320 J to 11,520 J. The resulting 1-year survival rate was 60% and the median survival time was 15 months, which is encouraging. Four patients survived over 24 months. The expected median survival time is 6–8 months for recurring malignant glioma. It is not known whether the extended survival time is due to patient selection or treatment efficacy.

Johansson et al. [45] did another I-PDT study using 5-ALA on 5 patients with non-resectable recurrent glioblastomas. Patients received 5-ALA orally at 20 or 30 mg/kg body weight 5–8 h before treatment. Light was delivered with four to six 0.6-mm-diameter cylindrical diffusers 20 or 30 mm long. Treatment light at 635 nm was provided at a constant power of 150 or 200 mW/cm for a total light dose of 720 J/cm per fiber. The maximum total light dose per patient ranged from 5700 J to 12,960 J. Tissue biopsies on three patients taken before light applications showed significant protoporphyrin IX concentrations in parts of the tumors. Protophorphyrin IX fluorescence was also observed from the tumors during treatment. These three patients responded favorably to the treatment, with survival time greater than 29 months. In two patients, no detectable protoporphyrin IX was found in the tumor biopsies and no detectable protoporphyrin IX fluorescence was observed during treatment. These two non-responders survived less than 9 months after treatment. The long-term survival of patients showing significant intratumoral protoporphyrin IX concentrations and intra-operative fluorescence was promising and indicates that protoporphyrin IX concentrations and fluorescence should be monitored before I-PDT begins to determine whether or not to proceed with the treatments.

Although survival results for some of the responders in the two studies appear promising, there were not enough patients in the studies or any results with randomized controls to determine if the responses are statistically significant. More trials with higher patient numbers are needed. In addition, treatment planning systems are being developed to aid I-PDT for neurosurgery [46] and may lead to greater improvement of treatment outcomes.

## 3. Summary

Multiple studies demonstrated that I-PDT could be utilized to treat deeply-seated and locally-advanced tumors. The light delivery from multiple fibers enables treatment of large tumors, which cannot be illuminated with EB-PDT. In the majority of patients, large tumors are associated with failure to respond to SOC therapy. The main advantage of I-PDT is that is can be safely administered in these patients. This treatment can be repeated multiple times, to provide local control. I-PDT is not associated with long-term toxicity, and can be delivered to tumors in the head and neck, pancreas. prostate and brain. Several research teams in the EU, US and Mexico demonstrated that I-PDT is a promising treatment options for these patients. The EMA approved the use of I-PDT with mTHPC for refractory LAHNC. The Cofepris, Mexico’s health authority, granted approval for I-PDT with Tookad^TM^ in the treatment of early-stage prostate cancer. The recent encouraging results from the Phase III study of I-PDT with Tookad^TM^ versus active surveillance in low-risk localized prostate cancer patients may lead to another approval [27]. Active surveillance was used as the comparator for the study, which is an acceptable method for patients with early stage prostate cancer.

In the US, I-PDT is being used in clinical studies to treat prostate cancer, primarily in patients that failed to respond to SOC therapy. One pilot study has been conducted in the treatment of LAHNC. However, no multicenter randomized trial of I-PDT versus SOC has been conducted in the US. We believe that such studies are required to gain approval from the US FDA (Food and Drug Administration). The research team at Roswell Park Cancer Institute (RPCI) is working towards that goal, with support from the National Cancer Institute at the US National Institute of Health. In this effort, the RPCI team is focused on optimizing the I-PDT for LAHNC in utilizing their treatment planning and real time dosimetry.

Several groups developed treatment planning and dosimetry systems to administer I-PDT [9,14,15,17,18,39,40,46,47,48,49]. These plans simulate light propagation in 3-D geometries mimicking the anatomy of target tumor and adjacent structures. The 3-D models were constructed from two-dimensional scans of CT, MRI or ultrasound. Cassidy et al. developed a general I-PDT treatment planning using Monte Carlo (MC) simulation method that is applicable to a broad range of study subjects, material properties, and lasers [48]. The method calculates the DVHs with a variance-reduction technique that reduces the number of packets used and the computational run time. In terms of dosimetry, the software simulates the light propagation dependent on tissue optical properties. The focus was on modeling the light propagation using either MC or FEM to solve the radiative transfer and diffusion equations, respectively. However, PDT includes not only light but also the PS photokinetic reactions and molecular oxygen to form singlet oxygen. A recent study of PDT dose metrics has shown that neither light intensity nor the product of light intensity and PS concentration sufficiently predicted tumor response in experimental animals. Instead, reacted singlet oxygen concentration proved to be the best dose metric for predicting outcomes [50].

In addition, many planning approaches assume that there are sufficient PS levels and oxygen in the target tumor, which may not always be true. We believe that in some cases this assumption may lead to under-treatment, thus, low PS and oxygen levels in the target tumor may result in no or partial response to I-PDT. While real-time fluorescence imaging and spectroscopy may help to assess the treatment efficacy, including the photochemical reaction (as underway by RPCI and Simphotek) in the modeling may further improve treatment guidance. In addition, pre- and post-imaging using CT with contrast may also be utilized to assess the response, as shown by researchers in I-PDT of pancreatic cancer [35].

We suggest that image-based treatment planning and real-time dosimetry are required to optimize and further advance the utilization of I-PDT. These tools will allow standardizing the treatment and support future multicenter trials needed to gain approval of I-PDT in the treatment of locally-advanced tumors.

PDT has been established as an alternate therapy for the treatment of various types of solid tumors. In addition, recent preclinical and clinical data suggest that PDT may have a role as an adjunct in cancer therapy [2,3,51,52,53]. Moreover, prior radiation therapy does not preclude the use of PDT to control malignant disease [54]. Thus, PDT and, in particular, I-PDT may be used as an additional therapy that has the potential to improve outcomes in patients with refractory or locally-advanced cancer who need better treatment options.

## Figures and Tables

**Table 1:** Table of representative photosensitizer (PS), light wavelengths and energy/intensity, number of subjects and general findings for interstitial photodynamic therapy (I-PDT) prostrate treatment. * approved in the US and EU for actinic keratosis, ** approved in Mexico for treating early-stage prostate cancer, *** approved in the EU for treating head and neck cancer. ALA: 5-aminolevulinic acid; MLu: motexafin lutetium; mTHPC: meso-tetrahydroxyphenylchlorin.

Drug	Drug Dose (mg/kg)	λ (nm)	Laser Settings	# of Patients	Results/Findings	Reference
ALA (*)	20	633	250 J/cm	14	Significant reduction in prostate-specific antigen (PSA) values was found.	Zack et al., 2003 [21]
MLu	2	732	150 J/cm	18	The 2 mg/kg MLu dose was found too low for effective treatment.	Verigos et al., 2006 [17]
MLu	2	732	150 mW/cm with 100 J/cm^2^ measured with isotropic detectors	3	Pilot study of diffuse reflectance spectroscopy (DRS) for tumor blood oxygenation and diffuse correlation spectroscopy (DCS) for tumor blood flow. Hemoglobin concentration decreased by 50% following I-PDT.	Yu et al., 2006 [22]
MLu	2	732	150 mW/cm	4	Simulations showed wide variation in light intensity in I-PDT treatment of prostate cancer.	Li and Zhu 2008 [9]
MLu	2	732	150 mW/cm	1	Numerical simulations demonstrate significant variation in optical properties in the target tumor.	Wang and Zhu 2009 [23]
Tookad^TM^ (**)	2	763	230–360 J/cm	6	Phase I study. Treatment was found to be safe and well tolerated.	Trachtenberg et al., 2007 [24]
Tookad^TM^	4,6	763	200–300 J/cm	4	Retrospective analysis of clinical trials to examine drug dose, energy fluence and time on I-PDT; Best result with 4 mg/kg and 200 J/cm.	Gross et al., 2003 [20]; Davidson et al., 2009 [15]; Betrouni et al., 2011 [19]
Tookad^TM^	4, 6	753	200J/cm	83	Negative biopsy after 6 months for 61/83 (74%); 4mg/kg and 200 J/cm were optimal for 38/46 (82.6%).	Azzouzi et.al., 2013 [25]
Tookad^TM^	2, 4, 6	753	200 J/cm	40	Phase II trial using 4 mg/kg activated with 753-nm light at a dose of 200 J/cm resulted in a treatment effect of 95% of the planned treatment volume in 12 men and negative biopsy after 6 months for 10/12 or 83.3%.	Moore et al., 2015 [26]
Tookad^TM^	4	753	150 mW/cm 200 J/cm	206/PDT, 207/active surveillance	Phase III trial; negative biopsy after 24 months in 49% (101) of patients who received PDT versus 14% (28) in the active surveillance group.	Azzouzi et. al., 2016 [27]
mTHPC (***)	0.15	652	100–150 mW	14	Phase I study, following radiotherapy treatment; partial gland was treated. Up to 91% necrosis or 49% necrosis if one lobe only; cited need for improved dosimetry.	Nathan et. al., 2002 [28]
mTHPC	0.15	652	100 J/cm	6	Early study, after 8–10 I-PDT treatments PSA level fell by 67%.	Moore et al., 2006 [29]
mTHPC	0.15	652	5 J/cm^2^ Calculated from lesion size measured with MRI	4	Online dosimetry, dose plans were provided with fiber positions and light dose was based on model; Results were that 5 J/cm^2^ was too low a light dose.	Swartling et al., 2010 [30]

**Table 2:** Representative I-PDT treatments for pancreatic cancers.

Drug	Drug Dose (mg/kg)	λ (nm)	Laser Settings	# of Patients	Results/Findings	Reference
mTHPC	0.15	652	100 mW per fiber; 20–40 J/cm	16	Tumors regrew at edges of necrotic regions. Median survival: 9.5 months.	Bown et al., 2002 [6]
Verteporfin	0.4	690	150 mW/cm; 5–40 J/cm per fiber	15	No necrosis at 5 J/cm; at 40 J/cm, necrosis was >12 mm in diameter; considerable variation depending on dose; median survival: 8.8 months.	Huggett et al., 2014 [35]

**Table 3:** Representative I-PDT treatments of head and neck cancer.

Drug	Drug Dose (mg/kg)	λ (nm)	Laser Settings	# of Patients	Results/Findings	Reference
mTHPC	0.15	652	100 mW/cm, 20 J/cm, flat cut	45	Median overall survival 14 months for responders (73%), versus 2 months for non-responders.	Lou et al., 2004 [37]
mTHPC	0.15	652	100 mW/cm, 20 J/cm, flat cut	14	Median overall survival 14 months.	Jager et al., 2005 [38]
mTHPC	0.15	652	200 J per site (10 mm) at 100 mW. Flat-cut fiber	21	Improvement in palliation (9/11), 60% overall survival after 45 months.	Jerjes et al., 2011 [7]
mTHPC	0.15	652	100 mW/cm, 30 J/cm, Cylindrical diffuser fiber	20	Median overall survival 15 months.	Karakullukcu et al., 2012 [8]

**Table 4:** Representative I-PDT treatments for brain cancers.

Drug	Drug Dose (mg/kg)	λ (nm)	Laser Settings	# of Patients	Results/Findings	Reference
ALA	20	633	Up to six cylindrical diffusers; total 4320–11,520 J (at 200 mW/cm)	10	Adult patients with recurrent malignant glioma; median survival 15 months.	Beck et al., 2007 [44]
ALA	20 or 30	635	4–6 cylindrical diffusers; total 5700–12,960 J; 720 J/cm (at 150-200 mW/cm)	5	Survival >29 months in three responders, <9 months in two non-responders.	Johansson et al., 2013 [45]

## Abbreviations

The following abbreviations are used in this manuscript:

PDT	photodynamic therapy
I-PDT	interstitial photodynamic therapy
RPCI	Roswell Park Cancer Institute
PS	photosensitizer
EB-PDT	external beam PDT
SOC	standard of care
MLu, Lutex	motexafin lutetium
Tookad^TM^	palladium bacteriopheophorbide
*mTHPC*, Foscan^TM^	meso-tetrahydroxyphenylchlorin
5-ALA	5-aminolevulinic acid
Verteporfin	Visudyne^TM^
3-D	three-dimensional
TRUS	transrectal ultrasound
PSA	Prostate Specific Antigen
BOLD	blood oxygenation level-dependent
MRI	magnetic resonance imaging
J	energy
J/cm^2^	energy density
J/cm	linear energy
mW/cm^2^	intensity
FEM	finite elements method
iDOSE	Interactive Dosimetry by Sequential Evaluation
MC	Monte Carlo
CT	computed tomography
LAHNC	locally advanced head and neck cancer
EMA	European Medicines Agency
